# Supplementary material for: Informing Patients about Biosimilar Medicines: The Role of European Patient Associations
Source: Pharmaceuticals (Basel). 2021 Feb 4;14(2):117. doi: 10.3390/ph14020117 (PMC7913743; doi:10.3390/ph14020117)
Supplement: Supplementary file 1 [file pharmaceuticals-14-00117-s001.zip › pharmaceuticals-1080408-final-SI/Supplementary Figure S1.docx]

**Supplementary Figure S1: PRISMA flow diagram of the literature review**

Studies included in qualitative synthesis
(n = 51)

Additional records included after snowballing

(n = 13)

Records excluded
(n = 1210)

Records identified through database searching
(n = 1769)

- PubMed (n = 540)
- Embase (n = 1229)

Records screened on title and abstract
(n = 1319)

Duplicates removed
(n = 450)

Full-text articles excluded
(n = 65)

Full-text articles assessed for eligibility
(n = 103)

## Identification

## Eligibility

## Included

## Screening
