# Supplementary material for: Informing Patients about Biosimilar Medicines: The Role of European Patient Associations
Source: Pharmaceuticals (Basel). 2021 Feb 4;14(2):117. doi: 10.3390/ph14020117 (PMC7913743; doi:10.3390/ph14020117)
Supplement: Supplementary file 1 [file pharmaceuticals-14-00117-s001.zip › pharmaceuticals-1080408-final-SI/Supplementary Table S1.docx]

**Supplementary Table S1: Structured literature search methodology**

| **Medline/PubMed** | | |
| --- | --- | --- |
| **A: Biosimilar medicines** | | |
| Concept | MeSH term | Free text (searched in title and abstract) |
| Biosimilar | “Biosimilar Pharmaceuticals” | “biosimilar”, “biosimilars”, “biosimilar agent”, “biosimilar agents”, “biosimilar pharmaceutical”, “biosimilar pharmaceuticals”, “similar biological medicinal product”, “similar biological medicinal products”, “follow-on biologic”, “follow-on biologics”, follow-on biological”, “follow-on biologicals”, “subsequent-entry biologic”, “subsequent-entry biologics”, “subsequent-entry biological”, “subsequent-entry biologicals”, “best-value biological”, “best-value biologicals”, “best-value biologic”, “best-value biologics” |
| Search query | "Biosimilar Pharmaceuticals"[Mesh] OR biosimilar[Title/Abstract] OR biosimilars[Title/Abstract] OR biosimilar-agent[Title/Abstract] OR biosimilar-agents[Title/Abstract] OR biosimilar-pharmaceutical[Title/Abstract] OR biosimilar-pharmaceuticals[Title/Abstract] OR similar-biological-medicinal-product[Title/Abstract] OR similar-biological-medicinal-products[Title/Abstract] OR follow-on-biologic*[Title/Abstract] OR subsequent-entry-biologic*[Title/Abstract] OR best-value-biologic*[Title/Abstract] | |
| **B: Education/information** | | |
| Concept | MeSH term | Free text (searched in title and abstract) |
| Education | “Education” | “education”, “training program*”, “educational activity”, “educational activities”, “learning”, “workshop*”, “literacy program*”, “training” |
| Information | “Information dissemination” | “information” |
| Communication | “Communication” | “communication” |
| Knowledge | “Knowledge” | “knowledge”, “attitude”, “attitudes”, “awareness”, “opinion”, “opinions”, “insight”, “insights” “belief”, “beliefs”, “perspective”, “perspectives” “view”, “views”, “understanding”, “perception”, “perceptions” |
| Search query | "Education"[Mesh] OR “Information dissemination”[MeSH] OR “Communication”[MeSH] OR “knowledge”[MeSH] OR “education”[tiab] OR “training-program”[tiab] OR “educational activity”[tiab] OR “educational activities”[tiab] OR “learning”[tiab] OR “workshop*”[tiab] OR “literacy program*”[tiab] OR “training”[tiab] OR “information”[tiab] OR “communication”[tiab] OR “knowledge”[tiab] OR “attitude”[tiab] OR “attitudes”[tiab] OR “awareness”[tiab] OR “opinion”[tiab] OR “opinions”[tiab] OR “insight”[tiab] OR “insights”[tiab] OR “belief”[tiab] OR “beliefs”[tiab] OR “perspective”[tiab] OR “perspectives”[tiab] OR “view”[tiab] OR “views”[tiab] OR “understanding”[tiab] OR “perception”[tiab] OR “perceptions”[tiab] | |
| **C: Patient** | | |
| Concept | MeSH term | Free text (searched in title and abstract) |
| Patient | “Patients” | “Patient”, “patients”, “sufferer”, “sufferers” |
| Search query | “Patients”[MeSH] OR “patient”[tiab] OR “patients”[tiab] OR “sufferer”[tiab] OR “sufferers”[tiab] | |

**Complete query Medline/PubMed:**

("Biosimilar Pharmaceuticals"[Mesh] OR biosimilar[Title/Abstract] OR biosimilars[Title/Abstract] OR biosimilar-agent[Title/Abstract] OR biosimilar-agents[Title/Abstract] OR biosimilar-pharmaceutical[Title/Abstract] OR biosimilar-pharmaceuticals[Title/Abstract] OR similar-biological-medicinal-product[Title/Abstract] OR similar-biological-medicinal-products[Title/Abstract] OR follow-on-biologic*[Title/Abstract] OR subsequent-entry-biologic*[Title/Abstract] OR best-value-biologic*[Title/Abstract]) AND ("Education"[Mesh] OR "Information dissemination"[MeSH] OR "Communication"[MeSH] OR “knowledge”[MeSH] OR "education"[tiab] OR "training-program"[tiab] OR "educational activity"[tiab] OR "educational activities"[tiab] OR "learning"[tiab] OR "workshop*"[tiab] OR "literacy program*"[tiab] OR "training"[tiab] OR "information"[tiab] OR "communication"[tiab] OR "knowledge"[tiab] OR "attitude"[tiab] OR "attitudes"[tiab] OR "awareness"[tiab] OR "opinion"[tiab] OR "opinions"[tiab] OR "insight"[tiab] OR "insights"[tiab] OR "belief"[tiab] OR "beliefs"[tiab] OR "perspective"[tiab] OR "perspectives"[tiab] OR "view"[tiab] OR "views"[tiab] OR "understanding"[tiab] OR "perception"[tiab] OR "perceptions"[tiab]) AND ("Patients"[MeSH] OR "patient"[tiab] OR "patients"[tiab] OR "sufferer"[tiab] OR "sufferers"[tiab])

| **Embase** | | |
| --- | --- | --- |
| **A: Biosimilar medicines** | | |
| Concept | Emtree term | Free text (searched in title and abstract) |
| Biosimilar | “Biosimilar agent” | ‘biosimilar’, ‘biosimilars’, ‘biosimilar-agent’, ‘biosimilar-agents’, ‘biosimilar-pharmaceutical’, ‘biosimilar-pharmaceuticals’, ‘similar-biological-medicinal-product’, ‘similar-biological-medicinal-products’, ‘follow-on-biologic’, ‘follow-on-biologics’, ‘follow-on-biological’, ‘follow-on-biologicals’, ‘subsequent-entry-biologic’, ‘subsequent-entry-biologics’, ‘subsequent-entry-biological’, ‘subsequent-entry-biologicals’, ‘best-value-biological’, ‘best-value-biologicals’, ‘best-value-biologic’ , ‘best-value-biologics’ |
| Search query | ‘biosimilar agent’/exp OR ‘biosimilar’:ti,ab,kw OR ‘biosimilars’:ti,ab,kw OR ‘biosimilar agent’:ti,ab,kw OR ‘biosimilar agents’:ti,ab,kw OR ‘biosimilar pharmaceutical’:ti,ab,kw OR ‘biosimilar pharmaceuticals’:ti,ab,kw OR ‘similar biological medicinal product’:ti,ab,kw OR ‘similar biological medicinal products’:ti,ab,kw OR ‘follow on biologic*’:ti,ab,kw OR ‘subsequent entry biologic*’:ti,ab,kw OR ‘best value biologic*’:ti,ab,kw | |
| **B: Information/education** | | |
| Concept | Emtree term | Free text (searched in title and abstract) |
| Education | “Education” | “education”, “training-program*”, “educational-activity”, “educational-activities”, “learning”, “workshop*”, “literacy-program*”, “training” |
| Information | “Information dissemination” | “information” |
| Communication | ‘Interpersonal communication’ | “communication” |
| Knowledge | “knowledge” | “knowledge”, “attitude”, “attitudes”, “awareness”, “opinion”, “opinions”, “insight”, “insights” “belief”, “beliefs”, “perspective”, “perspectives” “view”, “views”, “understanding”, “perception”, “perceptions” |
| Search query | ‘education’/exp OR ‘information dissemination’/exp OR ‘knowledge’/exp OR ‘interpersonal communication’/exp OR ‘education’:ti,ab,kw OR ‘training-program*’:ti,ab,kw OR ‘educational-activity’:ti,ab,kw OR ‘educational-activities’:ti,ab,kw OR ‘learning’ ‘workshop*’:ti,ab,kw OR ‘literacy-program*’:ti,ab,kw OR ‘training’:ti,ab,kw OR ‘information’:ti,ab,kw OR ‘communication’:ti,ab,kw OR ‘ knowledge’:ti,ab,kw OR ‘attitude’:ti,ab,kw OR ‘attitudes’:ti,ab,kw OR ‘awareness’:ti,ab,kw OR ‘opinion’:ti,ab,kw OR ‘opinions’:ti,ab,kw OR ‘insight’:ti,ab,kw OR ‘insights’:ti,ab,kw OR ‘belief’:ti,ab,kw OR ‘beliefs’:ti,ab,kw OR ‘perspective’:ti,ab,kw OR ‘perspectives’:ti,ab,kw OR ‘view’:ti,ab,kw OR ‘views’:ti,ab,kw OR ‘understanding’:ti,ab,kw OR ‘perception’:ti,ab,kw OR ‘perceptions’ | |
| **C: Patient** | | |
| Concept | Emtree term | Free text (searched in title and abstract) |
| Patient | “Patient” | “Patient”, “patients”, “sufferer”, “sufferers” |
| Search query | ‘patient’/exp OR ‘patient’ :ti,ab,kw OR ‘patients’:ti,ab,kw OR ‘sufferer’:ti,ab,kw OR ‘sufferers’:ti,ab,kw | |

**Complete query Embase:**

(‘biosimilar agent’/exp OR ‘biosimilar’:ti,ab,kw OR ‘biosimilars’:ti,ab,kw OR ‘biosimilar agent’:ti,ab,kw OR ‘biosimilar agents’:ti,ab,kw OR ‘biosimilar pharmaceutical’:ti,ab,kw OR ‘biosimilar pharmaceuticals’:ti,ab,kw OR ‘similar biological medicinal product’:ti,ab,kw OR ‘similar biological medicinal products’:ti,ab,kw OR ‘follow on biologic*’:ti,ab,kw OR ‘subsequent entry biologic*’:ti,ab,kw OR ‘best value biologic*’:ti,ab,kw) AND (‘education’/exp OR ‘information dissemination’/exp OR ‘knowledge’/exp OR ‘interpersonal communication’/exp OR ‘education’:ti,ab,kw OR ‘training-program*’:ti,ab,kw OR ‘educational-activity’:ti,ab,kw OR ‘educational-activities’:ti,ab,kw OR ‘learning’ ‘workshop*’:ti,ab,kw OR ‘literacy-program*’:ti,ab,kw OR ‘training’:ti,ab,kw OR ‘information’:ti,ab,kw OR ‘communication’:ti,ab,kw OR ‘ knowledge’:ti,ab,kw OR ‘attitude’:ti,ab,kw OR ‘attitudes’:ti,ab,kw OR ‘awareness’:ti,ab,kw OR ‘opinion’:ti,ab,kw OR ‘opinions’:ti,ab,kw OR ‘insight’:ti,ab,kw OR ‘insights’:ti,ab,kw OR ‘belief’:ti,ab,kw OR ‘beliefs’:ti,ab,kw OR ‘perspective’:ti,ab,kw OR ‘perspectives’:ti,ab,kw OR ‘view’:ti,ab,kw OR ‘views’:ti,ab,kw OR ‘understanding’:ti,ab,kw OR ‘perception’:ti,ab,kw OR ‘perceptions’) AND (‘patient’/exp OR ‘patient’:ti,ab,kw OR ‘patients’:ti,ab,kw OR ‘sufferer’:ti,ab,kw OR ‘sufferers’:ti,ab,kw)

**Inclusion criteria:**

- Published between 2006-2020
- Full text or conference abstract available
- Written in English
- Articles relevant for the European context
- Articles addressing one of the research questions below:

1. What kind of information is needed for patients about biosimilars?
2. How should patients be informed about biosimilar medicines?
3. How can patient information reach the patient effectively?
